# Patterns of plant organ-level non-structural carbohydrate content in response to nitrogen and phosphorus enrichment

**DOI:** 10.3389/fpls.2025.1659022

**Published:** 2025-09-30

**Authors:** Weiyi Zhou, Peirui Gu, Yuejuan Tang, Yuanming Zhang

**Affiliations:** ^1^ State Key Laboratory of Ecological Safety and Sustainable Development in Arid Lands, Xinjiang Institute of Ecology and Geography, Chinese Academy of Sciences, Urumqi, China; ^2^ Research Center for Ecology and Environment of Central Asia, Chinese Academy of Sciences, Urumqi, China; ^3^ University of Chinese Academy of Sciences, Beijing, China; ^4^ Independent Researcher, Urumqi, China

**Keywords:** nutrient enrichment, carbon storage, soluble sugar, mete-analysis, plant physiology

## Abstract

Carbon is one of the most crucial elements within plants, with its production and supply determining growth behaviors and physiological strategies. Nonstructural carbohydrates (NSC) serve as the “currency” of plant carbon flow, playing a key role in the balance between structural growth and carbon storage. However, the response patterns of NSC pools to varying concentrations and durations of nitrogen and phosphorus enrichment remain unclear. We conducted a meta-analysis compiling 1,313 independent data points from four plant organs-leaves, branches, stems, and roots-across global experiments to evaluate the impact of N and P enrichment on NSC pools in different organs. Our findings indicate that N limitation is widespread in ecosystems, whereas P limitation is not. Both the concentration and duration of N enrichment exhibit significant threshold effects on NSCs. Low to moderate levels of N enrichment led to varied increases in soluble sugar content (0.24% to 19.14%) and decreases in starch content (1.22% to 32.35%) in the leaves and branches of woody plants. However, this trend weakened or disappeared at high N concentrations. The NSC content in herbaceous plants was more sensitive to nutrient enrichment, with N enrichment significantly reducing their NSC reserves across all organs (by up to 90.72%). By integrating global data, this study not only addresses the gaps left by individual experiments in elucidating the spatio-temporal threshold responses of NSC to nutrient enrichment but also reveals the scarcity of studies on P addition and of long-term experiments in the existing literature. It reveals the growth-carbon storage strategies of plants under different nutrient conditions, contributing to biodiversity conservation and resource utilization in the context of future nitrogen deposition.

## 1 Introduction

Carbon is one of the most important elements in plants, and its production and supply determine their growth behavior and physiological strategies (Fatichi et al., 2019). Non-structural carbohydrates (NSC) are the primary “currency” of carbon flow in plants (Hoch and Körner, 2003). They are produced through photosynthesis and can serve as direct metabolic substrates or be converted into other energy-rich compounds, such as proteins and fatty acids, when needed (Hartmann and Trumbore, 2016). NSC acts as a carbon reservoir, providing a buffer when respiration, growth, and other physiological demands are not synchronized with photosynthesis (Prescott et al., 2020). Therefore, the size of the NSC pool can serve as an indicator of a plant’s growth status.

The two main components of NSC have distinct functions: soluble sugars are directly involved in physiological activities, while starch serves as a crucial energy reserve for future use (Hoch and Körner, 2003). These two components are highly interconvertible. The strategic balance between these two components reflects a plant’s carbon allocation strategy (Hartmann and Trumbore, 2016; Hoch and Körner, 2003). In this study, we define a “growth-driven mode” as a physiological state characterized by the mobilization of starch reserves to increase the pool of soluble sugars, thereby prioritizing immediate metabolic activity and structural growth over long-term storage. Conversely, we define a “stress-storage mode” as a conservative strategy involving the net accumulation of the total NSC pool, particularly starch, to build resilience against future uncertainties. Understanding how plants shift between these modes in response to nutrient enrichment is an important goal of our study. Moreover, the different carbon demands of plant organs result in significant differences in NSC pools across organs (Furze et al., 2019). For instance, in herbaceous plants, the largest NSC pool is typically found in the belowground parts, whereas in some woody plants, the largest NSC pool is located in the aboveground parts (Martínez-Vilalta et al., 2016). Given the importance of NSC to plants, many studies have explored how NSC responds to changes in nutrient availability (Jiang et al., 2022; Li et al., 2018, 2020; Ouyang et al., 2023). However, the mechanisms underlying the organ-level responses of NSC and its components to variations in nitrogen and phosphorus availability remain largely unexplored.

With the rapid development of global industrialization and urbanization, atmospheric nitrogen (N) deposition has increased three- to fivefold over the past century (Davidson, 2009; Yu et al., 2019), directly impacting soil and water bodies and profoundly influencing ecosystem functioning and plant metabolic capacity (Elser et al., 2007). Generally, nitrogen deposition promotes plant photosynthesis and nutrient transport (Wang et al., 2018; Zhang et al., 2018; Zhou W. et al., 2024). As the primary product of photosynthesis, carbohydrates are inevitably affected in terms of their forms and allocation within plant tissues under changing nitrogen availability (Wiley and Helliker, 2012). The effects of nitrogen nutrient status vary among species. Enhanced nitrogen availability has been shown to stimulate both carbon assimilation rates and biomass growth in two larch species, resulting in an increase in their NSC pools (Li et al., 2016). Another study found that nitrogen fertilization reduced the NSC pools across all organs of *Acer pseudoplatanus* L., while having no significant effect on the NSC content of *Abies alba* Mill. For *Picea abies* L., the NSC pool in aboveground organs exhibited a trend of initially decreasing and then increasing with higher nitrogen addition levels (Zhou X. et al., 2024). Nitrogen supplementation also influences carbon allocation strategies between plant organs. A meta-analysis compiling data from 75 experiments revealed that N enrichment decreased NSC concentrations in foliage (by ~5.4%) and roots (by ~5.0%), while simultaneously increasing them in above-ground wood (by ~6.1%) (Li et al., 2020). Additionally, the concentration of nitrogen added is a critical factor in these studies. Numerous studies indicate that nitrogen addition enhances plant growth up to a certain threshold (Nasto et al., 2019; Xiao et al., 2017; Zhang H. et al., 2020). At low nitrogen concentrations, carbon supply is positively affected, leading to an increase in the NSC pool (Wiley and Helliker, 2012). However, at high nitrogen concentrations, toxicity from excess salts can induce physiological drought, ultimately reducing the NSC pool (Liu et al., 2016). Interestingly, this threshold effect may be entirely reversed in some fast-growing species. In such cases, small amounts of N addition stimulate growth more than photosynthesis, resulting in carbon consumption rates exceeding carbon supply rates, which ultimately depletes the NSC pool (Li et al., 2020; Zhang Y.-L. et al., 2020). Conversely, high nitrogen concentrations suppress growth stimulation, slow carbon consumption, and thereby expand the NSC pool (Zhou X. et al., 2024).

Phosphorus plays a crucial role in photosynthesis, intracellular energy transfer, and carbohydrate transport (Warren, 2011; Zhang et al., 2014). While nitrogen availability primarily limits plant primary productivity, phosphorus mainly constrains energy exchange and phosphate synthesis within plants (Marklein and Houlton, 2012; Mo et al., 2019). Compared to studies on nitrogen addition, research on phosphorus nutrition is relatively scarce (Feng and Zhu, 2020). In some phosphorus-limited tropical forest ecosystems, leaf NSC concentrations are regulated by soil phosphorus availability rather than nitrogen availability (Liu et al., 2019; Mo et al., 2019; Walker and Syers, 1976). Mitigating phosphorus limitation can stimulate carbon consumption through growth metabolism, significantly reducing NSC content (Mo et al., 2020). In desert ecosystems, however, soil phosphorus content is typically high (Nasto et al., 2019), and long-term phosphorus addition can increase leaf phosphorus concentrations, leading to soil acidification, suppressing plant growth (Zhou W. et al., 2024), and ultimately impacting the NSC pool. In regions without phosphorus limitation, plants generally show weaker responses to phosphorus addition compared to nitrogen addition (Li et al., 2016). In summary, the effects of nitrogen and phosphorus enrichment on plant carbon supply and allocation are complex and lack a consistent overall pattern.

Although previous studies have revealed the response patterns of carbon supply, carbon storage, or hydraulic traits at the organ level to nitrogen enrichment (Li et al., 2018, 2020; Liu et al., 2016; Zhang et al., 2018), research on the effects of phosphorus enrichment on plant carbon allocation remains scarce. Furthermore, past studies have often overlooked the influence of nutrient addition concentrations and durations, limiting our understanding of the threshold effects of nutrient addition on carbon supply and storage. To address these gaps, we compiled 1,313 individual data points from 79 species and conducted a meta-analysis to evaluate the general patterns of nitrogen and phosphorus enrichment on plant carbon storage. Our study aims to answer the following scientific questions: (1) How do different concentrations of nitrogen and phosphorus additions affect NSC content? (2) How does the duration of nutrient treatments influence NSC content? (3) What are the differences in these effects across different plant organs? Our ultimate goal is to uncover the carbon strategies of plants in response to changes in nitrogen and phosphorus availability, contributing to biodiversity conservation and resource management under future global nitrogen deposition scenarios.

## 2 Materials and methods

### 2.1 Data compilation

**Figure 1:**
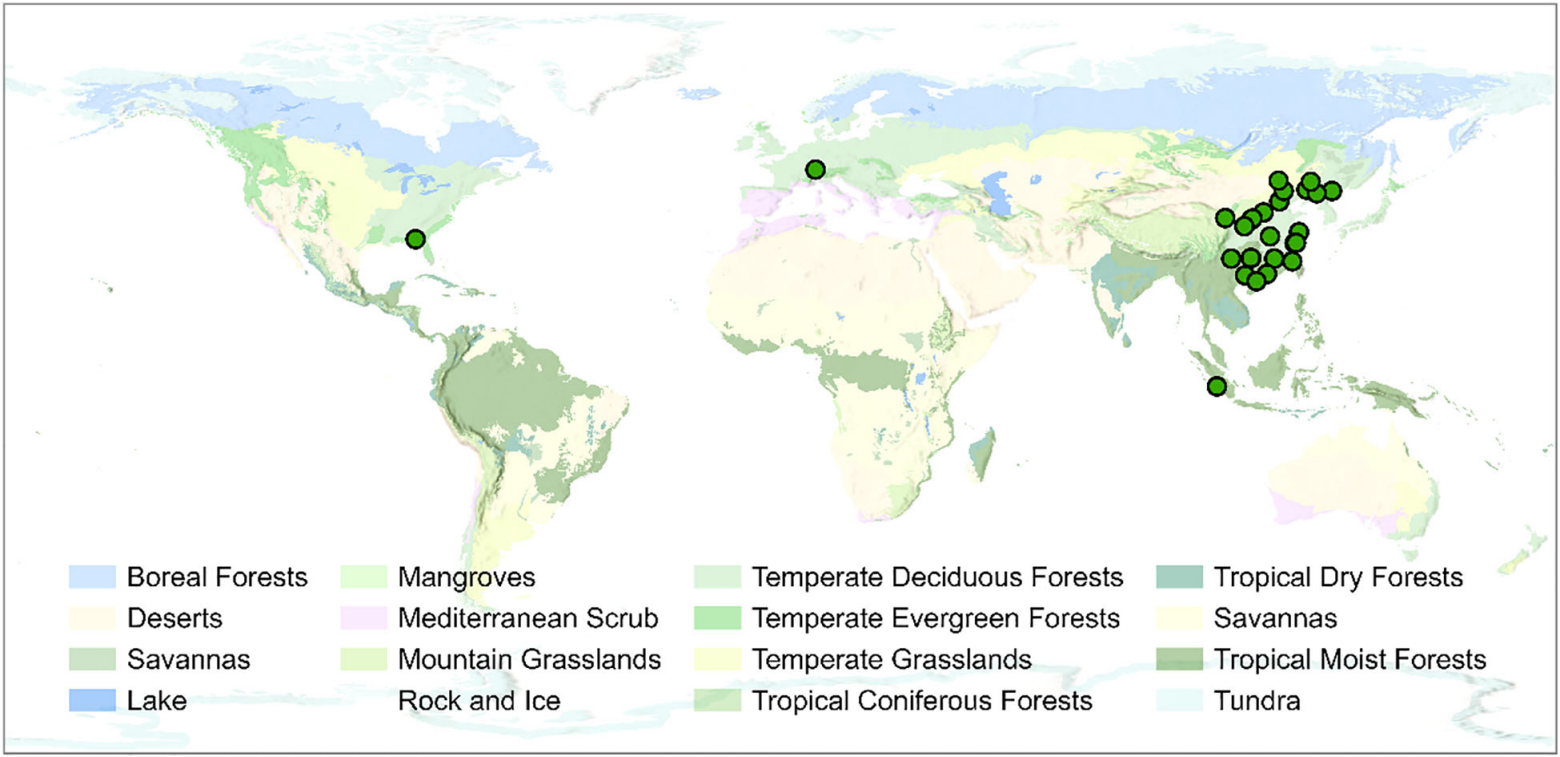
Global distribution of study sites used in the meta-analysis (green dots) overlaid on the terrestrial biome classification map.

We conducted a literature search in the Web of Science resource for journal articles published between January 2000 and December 2023, using the following keywords: “nonstructural carbohydrate*” or “NSC” or “TNC” or “soluble sugar” and “fertiliz*” or “nutrition*” or “nitrogen*” or “ phosphorus”. This search identified a total of 13,866 articles. The selection criteria for the included studies were as follows: (1) The studies must be based on manipulation experiments with control and experimental groups, with research subjects being either woody or herbaceous plants. (2) Only field-based nutrient addition studies were included, excluding laboratory incubation studies. (3) The fertilizers must contain only nitrogen (N), phosphorus (P), or both nitrogen and phosphorus, with no other nutrients. (4) The studies must provide both means and sample sizes for the reported data. Ultimately, 30 studies were selected for inclusion in our dataset (see **Supplementary File** for details), with their geographic distribution shown in **Figure 1**. The dataset comprises 62 plant species and 1,313 independent organ-level observations of NSCs. The content of different NSC components across various plant organs are presented in **Supplementary Figures S1** and **S2**.

From these articles, we extracted the NSC, soluble sugar, and starch contents in leaves, branches, stems, and roots, and standardized the units to mg/g. Additionally, we recorded research species, type of fertilizer, nitrogen and phosphorus addition rates, experimental duration, geographical location, leaf habit, and other relevant background information. Data were either directly retrieved from tables provided in the articles or extracted from images using WebPlotDigitizer software (He et al., 2020).

### 2.2 Data categorization and standardization

For each study, we prioritized the original classification of fertilization intensity if provided in the publication. If a study did not supply a classification, we standardized the fertilization amounts to grams per hectare per year (g/ha yr). Based on the overall distribution of application rates across our dataset, we defined concentrations as low (< 50 g/ha yr), medium (50–100 g/ha yr), and high (> 100 g/ha yr). While formal methods like breakpoint analysis can identify precise thresholds, they require a consistent continuous predictor, which was unavailable due to our hierarchical approach of honoring original study classifications. Therefore, our categorical analysis represents a robust strategy for identifying general patterns from diverse experimental designs, aligning with the primary goal of this synthesis.

Furthermore, we classified fertilization durations as short-term (≤ 6 months), medium-term (> 6 months to ≤ 3 years), and long-term (> 3 years). If a study reported data at multiple sampling dates, we utilized the data corresponding to the actual sampling times. Finally, due to limited data for finer classifications, we analyzed plant life forms based on two broad categories: herbaceous and woody plants, which are known to differ significantly in their carbon supply and storage strategies.

### 2.3 Meta analysis

The natural logarithm of the response ratio represents the effect size, assessing the relative change between the nutrient addition treatment and the control (Li et al., 2020). The formula for calculating the log response ratio is as follows (Equation 1):


(1) $$\ln\left(\text{RR}\right)=\ln\left(\frac{{\bar{X}}_{t}}{{\bar{X}}_{c}}\right)=\ln{\bar{X}}_{t}-\ln{\bar{X}}_{c}$$

where ${\bar{X}}_{t}$ is the mean NSC value in the treatment and ${\bar{X}}_{c}$ is that in the control (He et al., 2020; Liu et al., 2016).

The formula for calculating the variance of each effect value is as follows (Equation 2):


(2) $$v=\frac{S_{t}^{2}}{n_{t}\;{\bar{X}}_{t}^{2}}+\frac{S_{c}^{2}}{n_{c}\;{\bar{X}}_{c}^{2}}$$

where $n_{t}$ and $n_{c}$ are the sample sizes for the treatment and control groups, respectively, and $S_{t}$ and $S_{c}$ denote their corresponding standard deviations. ${\bar{X}}_{t}$ and ${\bar{X}}_{c}$ are the mean values of the variable in the treatment and control groups, respectively.

The weighting factor ( w ) for each observation is calculated by the inverse of the variance ( v ) (Equation 3):


(3) $$w=\frac{1}{v}$$

If a study includes multiple results for a single variable, we adjusted the weights based on the total number of observations in each study to reduce the influence of repeated measurements from the same site. The final weighting factor ( $w^{\prime }$ ) and effect size ( $\ln\bar{RR}$ ) were calculated using the following formulas (Equations 4, 5):


(4) $$w^{\prime }=\frac{W}{n}$$

(5) $$\ln\bar{RR}=\frac{\sum_{i}\left(w_{i}^{'}\times \ln RR_{i}\right)}{\sum_{i}w_{i}^{'}}$$

The distributions of all effect size results are shown in **Supplementary Figures S3** and **S4**. If the 95% confidence interval (CI) for a variable overlap with zero, it indicates that nutrient addition has no significant effect on the variable. Otherwise, the effect is considered statistically significant. The percentage change relative to the control was calculated as (Equation 6):


(6) $$e^{\ln\bar{RR}}\;-\;1$$

We conducted a meta-analysis using the “metafor” package in R 4.4.1 with the REML method. Data following a normal distribution were analyzed using one-way ANOVA followed by Tukey’s HSD method, while data not following a normal distribution were analyzed using the Kruskal-Wallis test followed by Dunn’s test.

## 3 Results

### 3.1 Effect of nitrogen enrichment on the content of NSC and its components

**Figure 2:**
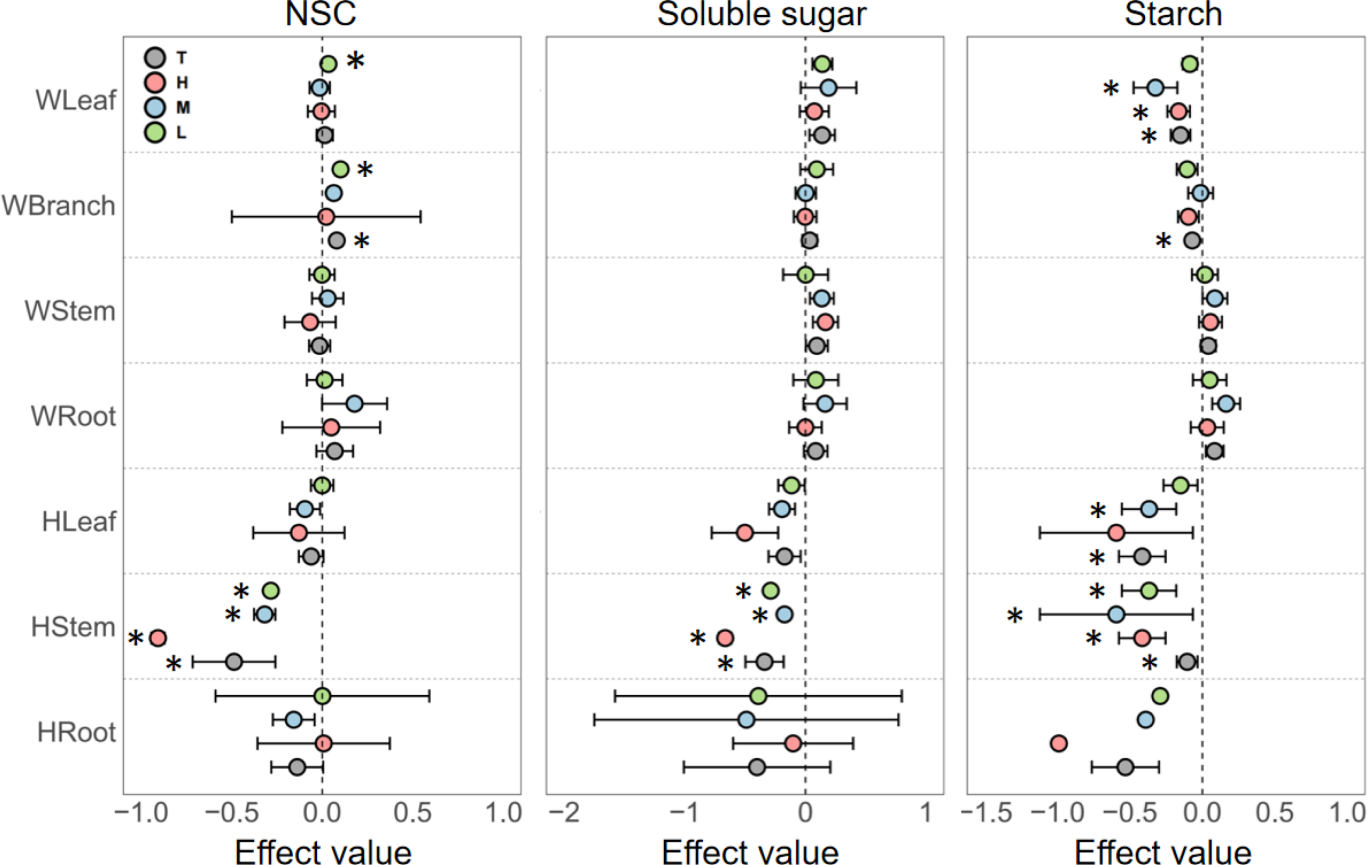
Responses of non-structural carbohydrates (NSC), soluble sugars, and starch in different organs of woody and herbaceous plants to various N addition levels. T, the pooled effect across all treatment levels; H, high nutrient addition; M, medium nutrient addition; L, low nutrient addition. The “W” before organ categories on the y-axis represents woody plants, and “H” represents herbaceous plants. Error bars indicate the standard error of effect sizes. See **Supplementary Table S1** for quantified percentage changes and **Supplementary Figure S5** for effect size distributions. *Indicates a significant difference from the control group (95% CI of the effect size does not overlap with zero).

The effects of nitrogen addition on the NSC and soluble sugar contents in woody plant leaves were minor, with a significant increase in NSC pool observed only under low nitrogen addition. However, nitrogen addition significantly decreased starch content in leaves across all concentrations. Notably, the magnitude of this negative effect was greatest at low nitrogen levels and diminished as the concentration increased. In branches, nitrogen addition had a significant positive effect on NSC content but a significant negative effect on starch content. For woody plant stems and roots, nitrogen addition generally had a positive effect on NSC and its components, but the effects were not statistically significant (**Figure 2**).

In contrast, herbaceous plants showed a significantly stronger response to nitrogen addition, predominantly resulting in decreased NSC content. Specifically, in leaves, NSC, soluble sugar, and starch contents decreased progressively with increasing nitrogen levels. In stems, nitrogen enrichment at all concentrations significantly reduced NSC content. In roots, while NSC content generally decreased, the changes exhibited high variability and uncertainty (**Figure 2**).

### 3.2 Effect of phosphorus enrichment on the content of NSC and its components

**Figure 3:**
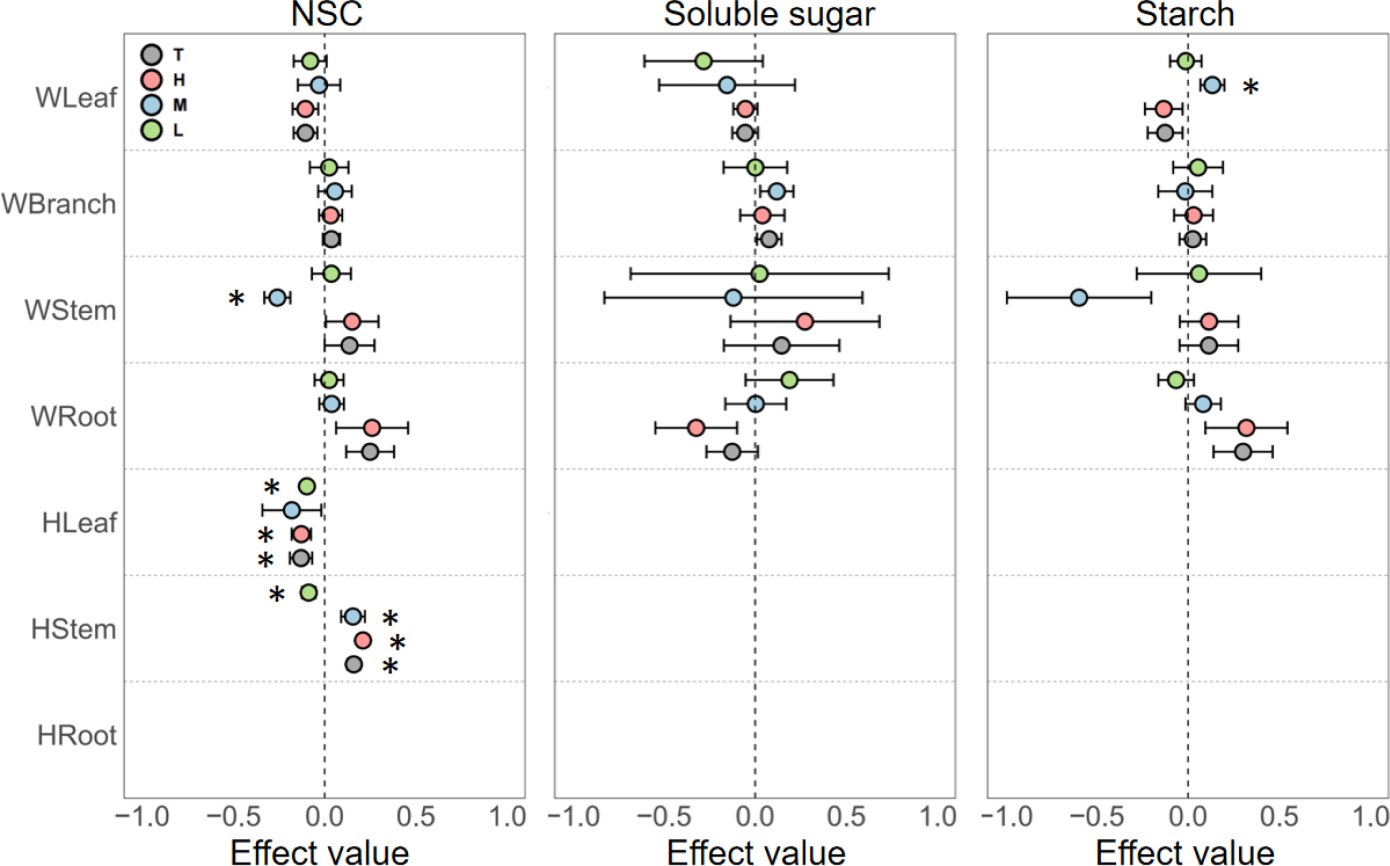
Responses of NSC, soluble sugars, and starch in different organs of woody and herbaceous plants to various P addition levels. T, the pooled effect across all treatment levels; H, high nutrient addition; M, medium nutrient addition; L, low nutrient addition. The “W” before organ categories on the y-axis represents woody plants, and “H” represents herbaceous plants. Error bars indicate the standard error of effect sizes. See **Supplementary Table S2** for quantified percentage changes and **Supplementary Figure S6** for effect size distributions. *Indicates a significant difference from the control group (95% CI of the effect size does not overlap with zero).

Phosphorus enrichment had highly variable effects on the NSC content of different organs in woody plants. For leaves, phosphorus enrichment generally exerted a negative effect on NSC and its components, with the exception of medium-level phosphorus enrichment, which significantly increased starch content. In branches, phosphorus enrichment generally increased NSC content, but none of these effects were statistically significant. In stems, the effects of phosphorus enrichment on NSC content fluctuated significantly, showing a trend of promotion at low levels, suppression at medium levels, and promotion again at high levels. In roots, NSC and starch contents increased with increasing phosphorus concentrations, while soluble sugar content decreased as phosphorus concentration increased (**Figure 3**).

Due to the scarcity of experimental data on the response of herbaceous plant NSC content to phosphorus enrichment, our analysis only allowed for discussion of NSC responses in certain herbaceous plant organs. In leaves, phosphorus enrichment at all concentrations reduced NSC content. For stems, low-level phosphorus enrichment reduced NSC content, but as phosphorus concentration increased to medium or high levels, the effect shifted from suppression to promotion (**Figure 3**).

### 3.3 Trends of NSC content under different nitrogen enrichment durations

**Figure 4:**
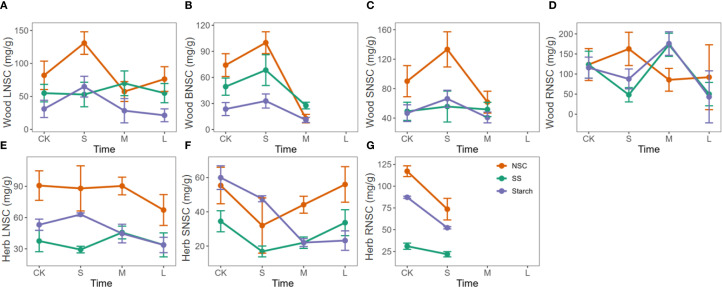
Response of NSC, soluble sugars, and starch to the duration of nitrogen enrichment in different organs of woody and herbaceous plants. The prefixes L, B, S, and R before NSC denote Leaf, branch, stem, and root, respectively. **(A–D)** Influence of N enrichment duration on NSC and its component contents in the leaves, branches, stems, and roots of woody plants. **(E–G)** Influence of N enrichment duration on NSC and its component contents in the leaves, branches, stems, and roots of herbaceous plants. The data for each nutrient addition duration were weighted based on sample size, with larger sample sizes contributing greater weight to the corresponding nutrient treatment duration in each organ. Fertilization duration was categorized into three levels: short-term (S,< 6 months), medium-term (M, > 6 months and ≤ 3 years), and long-term (L, > 3 years), based on standardized metadata from the original studies. The orange, green, and purple lines represent the responses of total NSC, soluble sugars (SS), and starch, respectively.

Nitrogen enrichment in woody plants generally showed a trend of initial promotion followed by suppression for NSC, soluble sugars, and starch contents in leaves, branches, and stems. Short-term nitrogen treatments significantly increased NSC content, but as the treatment duration extended to one year or more, this promotive effect diminished and even turned inhibitory. In roots, the trend differed slightly, with NSC content showing a similar initial increase and subsequent decrease, whereas soluble sugars and starch exhibited a pattern of initial decline, followed by an increase, and then another decrease (**Figures 4A-D**).

In herbaceous plants, nitrogen enrichment had little to no promotive effect on NSC accumulation. In leaves, NSC content gradually decreased with prolonged treatment. In stems, NSC content dropped significantly at first and then slowly recovered over time (**Figures 4E-G**).

### 3.4 Trends of NSC content under different phosphorus enrichment durations

**Figure 5:**
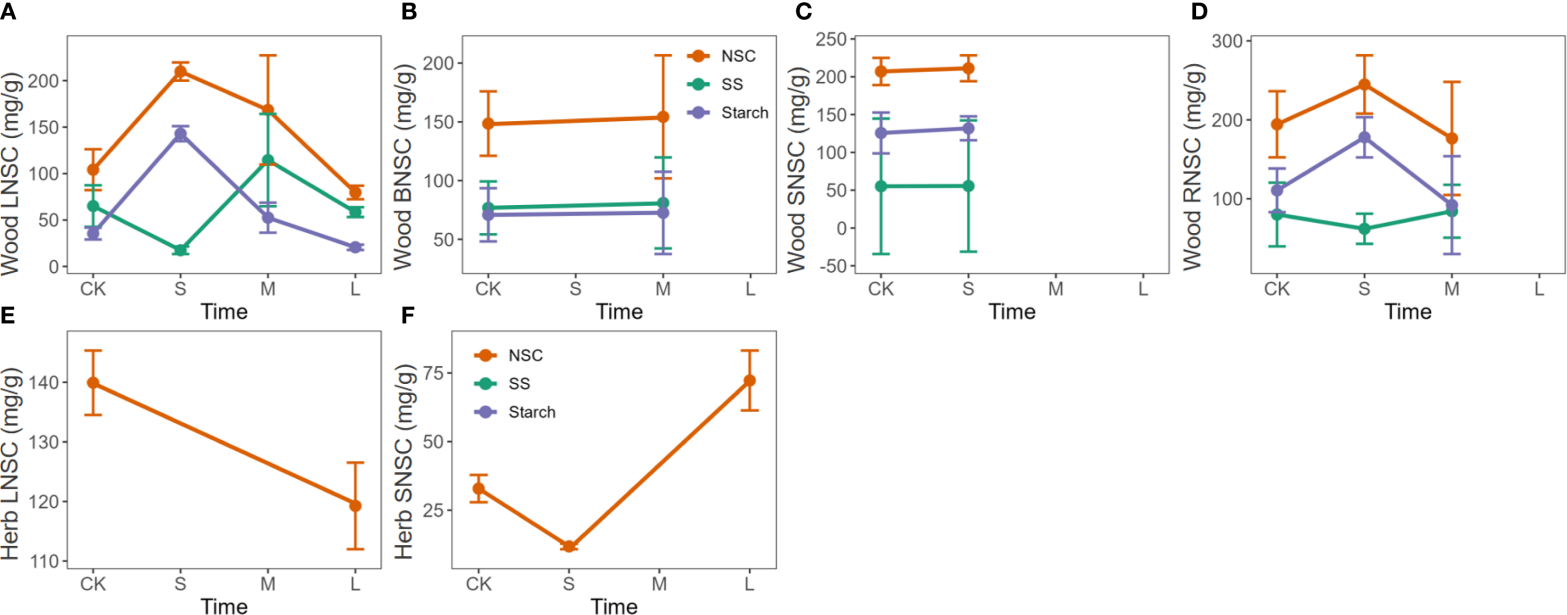
Response of NSC, soluble sugars, and starch to the duration of phosphorus enrichment in different organs of woody and herbaceous plants. The prefixes L, B, S, and R before NSC denote Leaf, branch, stem, and root, respectively. **(A–D)** Influence of P enrichment duration on NSC and its component contents in the leaves, branches, stems, and roots of woody plants. **(E, F)** Influence of P enrichment duration on NSC and its component contents in the leaves, branches, stems, and roots of herbaceous plants. Fertilization duration was categorized into three levels: short-term (S,< 6 months), medium-term (M, > 6 months and ≤ 3 years), and long-term (L, > 3 years), based on standardized metadata from the original studies. The orange, green, and purple lines represent the responses of total NSC, soluble sugars (SS), and starch, respectively. Data for SS and starch in herbaceous plants (panels e and f) were insufficient to perform a robust trend analysis of the duration effect and are therefore not shown.

Phosphorus enrichment showed an initial promotive effect followed by suppression on NSC and starch contents in the leaves and roots of woody plants, while soluble sugar content exhibited a reverse trend of initial suppression followed by promotion. However, no significant trends were observed in the NSC content of branches and stems (**Figures 5A-D**). In herbaceous plants, prolonged phosphorus enrichment significantly reduced NSC content in leaves, while NSC content in stems showed a trend of initial decrease followed by an increase (**Figures 5E, F**).

## 4 Discussion

### 4.1 Threshold effects are prevalent in the effects of nitrogen enrichment on NSC content

As is well known, nitrogen is a critical element in the construction of chloroplasts, and nitrogen availability is a key limiting factor for photosynthesis (Yue et al., 2017). Nitrogen enrichment can alleviate nitrogen limitation in the local soil, optimize the physiological state and structural integrity of leaves, and enhance photosynthetic activity (Borghetti et al., 2017; Hacke et al., 2010; Zhang et al., 2018). Consequently, the non-structural carbohydrate (NSC) pool enters a state where supply exceeds consumption, leading to an increase in NSC content. Generally, NSC transport adheres to the principle of proximal transport, moving from the upper morphological regions to the lower ones (Dietze et al., 2014; Tixier et al., 2018). As the primary site for NSC production (Chapin et al., 1990), leaves are the most directly and significantly affected by nitrogen enrichment. Nitrogen enrichment induces plants to adopt a “growth-driven mode,” increasing carbon allocation towards respiration and structural growth. Soluble sugars, which serve as direct substrates for respiratory and other metabolic activities (Gibon et al., 2009), are rapidly consumed in this “growth-driven mode.” However, their overall content still increases due to the substantial breakdown of starch replenishing the soluble sugar pool (Hoch and Körner, 2003). Therefore, the specific pattern of NSC component changes involves the accumulation of soluble sugars in the leaves, while starch is significantly depleted (**Figure 2**; **Supplementary Figure S3**). Similar results have been observed in studies on *Quercus mongolica* (Zhang Y.-L. et al., 2020). However, this pattern is not constant across all growth stages. When the study subjects are seedlings, nitrogen enrichment drives them into a more pronounced “growth-driven mode,” leading to the rapid consumption of all NSC components, resulting in a reduced NSC pool and accelerated biomass growth (Li et al., 2020).

Downward in the plant, the distribution patterns of NSC and its components in branches and stems are similar to those in leaves, but the magnitude of changes gradually decreases with increasing distance from the carbon source organs (leaves). Notably, the NSC content in leaves and branches, as well as leaf starch content under medium or low nitrogen enrichment, exhibit very clear “growth-driven mode” carbon allocation strategies. However, when nitrogen enrichment concentrations reach high levels, this strategy disappears, supporting our hypothesis that the impact of nitrogen enrichment on the NSC pool exhibits a threshold effect (Du et al., 2020; Zhou X. et al., 2024; Zhu et al., 2021). In contrast, the NSC variation pattern in roots is entirely different from that in other organs. Under nitrogen enrichment, the NSC pools and their components in roots are expanded. Subterranean NSC reserves are crucial for tree recovery from stress (He et al., 2020), serving as energy sources for growth and development following adverse conditions such as drought, thereby ensuring the energy balance of the trees (Hartmann et al., 2013). Thus, under nitrogen enrichment, the aboveground parts adhere to a “growth-driven” carbon allocation model, while the underground parts follow a “stress-storage” carbon allocation model.

Recent nitrogen addition experiments on three tree species have demonstrated that, for *Acer pseudoplatanus*, nitrogen enrichment initially increases and subsequently decreases its NSC content (Zhou X. et al., 2024). Another study similarly found that nitrogen addition first promotes and then inhibits NSC content in *Syzygium bullockii* and *Carallia brachiate* (Chen et al., 2022). Researchers typically focus on the seasonal variations of NSC content (Martínez-Vilalta et al., 2016) and NSC dynamics under prolonged drought stress (Rowland et al., 2015), paying less attention to the temporal threshold effects of nutrient enrichment. Consequently, studies addressing these threshold effects are rare and valuable. Our meta-analysis, integrating various studies as hypothesized, revealed that the duration of nitrogen enrichment also exhibits a significant threshold effect on NSC.

### 4.2 Phosphorus is not a major limiting factor for NSC pools expansion

Firstly, no significant promoting effect of phosphorus enrichment on the NSC pool was observed (**Figure 3**; **Supplementary Figure S4**). Alleviating phosphorus limitation did not drive plants into a growth-driven mode as nitrogen alleviation does, indicating that phosphorus limitation is not as prevalent in ecosystems as nitrogen limitation. A long-term experiment also yielded similar results, where phosphorus addition slowed plant growth rates and prompted more conservative resource allocation strategies (Báez and Homeier, 2018). Unlike nitrogen, which directly mediates plant photosynthetic processes (Fleischer et al., 2013) and thereby influences the NSC pool (Wiley and Helliker, 2012), phosphorus affects the NSC pool indirectly. Consequently, the gradient effect from the upper to the lower parts of the plant, as seen with nitrogen enrichment, was not observed with phosphorus enrichment. Instead, fluctuations in NSC content were markedly greater in stems and roots. Phosphorus fertilization can stimulate xylem growth and reduce conduit wall thickness (Thomas et al., 2006; Yi et al., 2022). Rapidly growing xylem with thinner conduit walls and pit membranes allows small air bubbles to pass through more easily, thereby increasing the likelihood of xylem embolism (Cochard et al., 2010; Delzon et al., 2010). Stems and roots are the largest biomass organs in woody plants and have the longest water transport distances (Cochard et al., 2010). Different species exhibit significant variations in their tolerance to phosphorus effects, as well as in the conditions governing water transport efficiency and safety (Villagra et al., 2013). Therefore, the indirect effect of phosphorus variation on NSC content through the regulation of water transport is most pronounced in these two organs.

Notably, as phosphorus enrichment concentrations increase, soluble sugars in leaves exhibit a clear upward trend, whereas the opposite trend is observed in roots (**Figure 2**). The reason for this is twofold. With higher phosphorus enrichment concentrations, soil water potential increases, leading plants to experience physiological drought (Hasanuzzaman et al., 2018) and necessitating the accumulation of soluble sugars to regulate internal osmotic pressure (Secchi and Zwieniecki, 2011). Meanwhile, aboveground parts, especially the canopy, serve as the primary sites for carbon assimilation and fruit development, thereby possessing a higher priority for carbon allocation (Minchin and Thorpe, 1996). Soluble sugars, acting as carbon currency, are transported from underground to aboveground parts, resulting in a negative correlation between soluble sugar contents in leaves and roots. In summary, the effects of nitrogen and phosphorus enrichment exhibit significant differences in woody plants.

### 4.3 Differences in nutrient enrichment in woody and herbaceous plants

Herbaceous plants respond to nutrient enrichment in their NSC content in a manner that is completely different from woody plants. Herbaceous plants possess smaller biomass and are typically annual or biennial (Liu et al., 2016). Additionally, herbaceous plants are characterized by rapid growth and weaker physiological regulatory capabilities (Zhao and Dixon, 2011), making them highly sensitive to environmental changes, including nutrient enrichment. Studies have demonstrated that nitrogen addition significantly affects the richness, diversity, and stoichiometric ratios of herbaceous plants (Miao et al., 2024; Soons et al., 2017; Wu et al., 2021). In this context, the growth-promoting effects of nitrogen enrichment are more pronounced in short-lived herbaceous plants, resulting in extensive consumption of NSC for growth. This is evidenced by a substantial reduction in NSC pools and their components across almost all concentrations of nitrogen enrichment, with NSC and its component contents decreasing as nitrogen enrichment concentration increases (**Figure 2**).

## 5 Conclusion and outlook

Our results demonstrate that nitrogen limitation is widespread in ecosystems. Nitrogen enrichment affects the NSC content in the aboveground parts of woody plants in a stepwise decreasing manner from top to bottom, with both the concentration and duration of nitrogen enrichment exhibiting significant threshold effects on NSCs. Moderate to low levels of nitrogen enrichment induce a “growth-driven mode” in the aboveground parts of plants, characterized by a substantial decrease in starch content and an increase in soluble sugars. However, at high nitrogen concentrations, this mode disappears. In contrast, the underground parts of woody plants adopt a conservative carbon storage mode, with nitrogen enrichment leading to an expansion of the NSC pool. NSC content in herbaceous plants is more sensitive to nutrient enrichment; nitrogen enrichment prompts rapid growth in herbaceous plants, significantly reducing their NSC pools. Phosphorus enrichment does not promote the expansion of the NSC pool and affects NSCs indirectly through metabolic activities and water transport, etc.

However, it is noteworthy that due to the scarcity of related studies and limitations in data quantity, we were unable to discuss whether a threshold effect of phosphorus enrichment exists on the NSC pools of herbaceous plants. Additionally, in examining the duration of nutrient enrichment, we did not categorize nutrient enrichment concentrations but rather synthesized them as a whole. Precisely by integrating these past studies, we were able to identify key gaps in the field, such as the lack of research comparing the effects of high-concentration, long-duration versus low-concentration, long-duration nitrogen enrichment. Future work can now build upon our results to further disentangle the interactive effects of nutrient concentration and duration. In summary, our study not only elucidates the growth-carbon storage strategies of plants under different nutrient conditions but also provides a foundational framework that will guide the next wave of research, contributing to biodiversity conservation and resource utilization in the context of future nitrogen deposition.

## Data Availability

The original contributions presented in the study are included in the article/**Supplementary Material**. Further inquiries can be directed to the corresponding authors.
