# Supplementary material for: Patterns of plant organ-level non-structural carbohydrate content in response to nitrogen and phosphorus enrichment
Source: Front Plant Sci. 2025 Sep 30;16:1659022. doi: 10.3389/fpls.2025.1659022 (PMC12518271; doi:10.3389/fpls.2025.1659022)
Supplement: Supplementary file 1 [file DataSheet1.docx]

**Supplementary information**


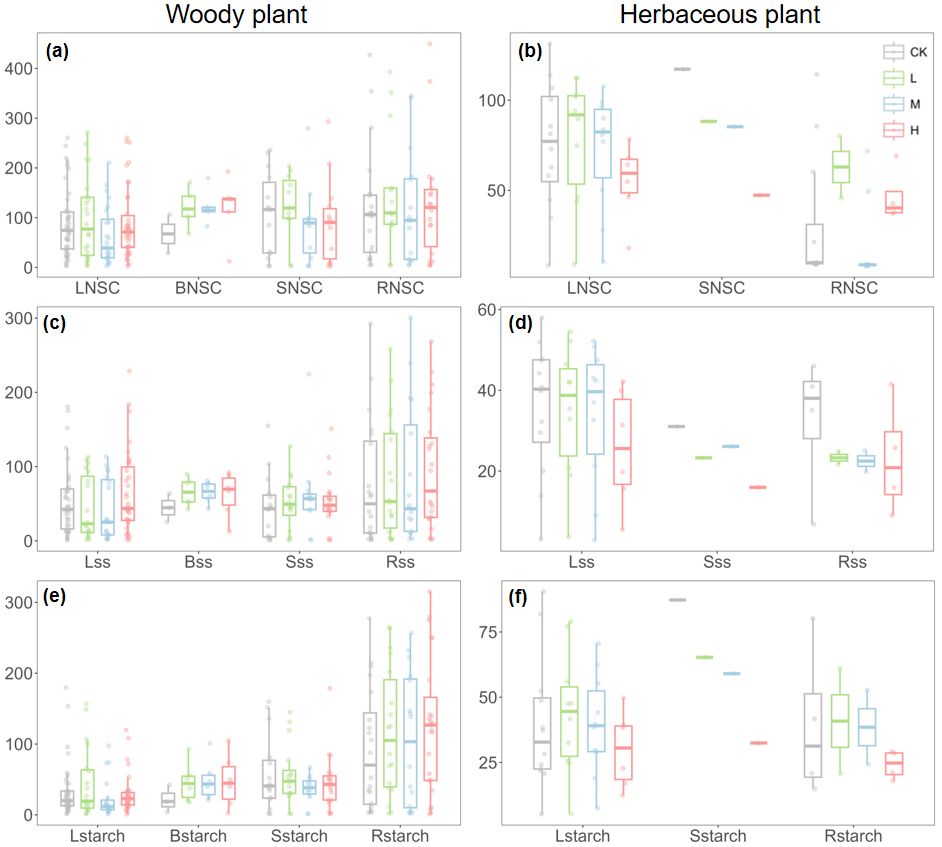


**Figure S1.** Nonstructural carbohydrate (NSC), soluble sugar (SS) and starch contents at different nitrogen enrichment concentrations. The prefixes L, B, S, and R before NSC denote Leaf, branch, stem, and root, respectively.


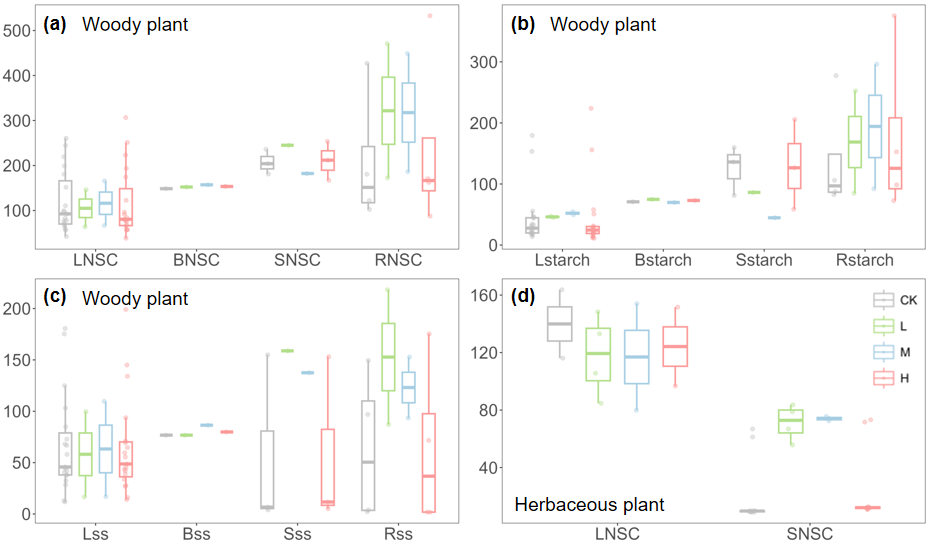


**Figure S2.** NSC, SS and starch contents at different phosphorus enrichment concentrations. The prefixes L, B, S, and R before NSC denote Leaf, branch, stem, and root, respectively.

**Table S1** Effects values of different N enrichment on NSCs in different woody and herbaceous plant organs.

|  |  |  | Effect values (%) | |  |  |
| --- | --- | --- | --- | --- | --- | --- |
| Life forms | Organ | N level | NSC | Soluble sugar | | Starch |
| Woody | Leaf | Low | 3.45 | 13.99 | | -8.64 |
| Woody | Leaf | Medium | -1.38 | 19.14 | | -32.35 |
| Woody | Leaf | High | -0.45 | 7.34 | | -16.38 |
| Woody | Leaf | Total | 1.44 | 13.85 | | -15.04 |
| Woody | Branch | Low | 10.15 | 9.33 | | -10.47 |
| Woody | Branch | Medium | 6.4 | 0.24 | | -1.22 |
| Woody | Branch | High | 2.25 | -0.25 | | -9.6 |
| Woody | Branch | Total | 8.04 | 3.46 | | -6.97 |
| Woody | Stem | Low | -0.12 | 0.15 | | 1.77 |
| Woody | Stem | Medium | 3.02 | 13.57 | | 8.65 |
| Woody | Stem | High | -6.66 | 16.58 | | 5.6 |
| Woody | Stem | Total | -1.42 | 9.46 | | 4.13 |
| Woody | Root | Low | 1.33 | 8.6 | | 5.07 |
| Woody | Root | Medium | 17.88 | 16.3 | | 16.42 |
| Woody | Root | High | 5.01 | -0.03 | | 3.37 |
| Woody | Root | Total | 6.92 | 8.47 | | 8.47 |
| Herbaceous | Leaf | Low | -0.01 | -11.56 | | -15.05 |
| Herbaceous | Leaf | Medium | -9.55 | -19.44 | | -36.76 |
| Herbaceous | Leaf | High | -12.87 | -50.17 | | -59.35 |
| Herbaceous | Leaf | Total | -6.11 | -17.31 | | -41.5 |
| Herbaceous | Stem | Low | -28.4 | -28.85 | | -36.76 |
| Herbaceous | Stem | Medium | -31.77 | -17.33 | | -59.35 |
| Herbaceous | Stem | High | -90.72 | -66.52 | | -41.5 |
| Herbaceous | Stem | Total | -48.7 | -33.91 | | -10.47 |
| Herbaceous | Root | Low | 0.12 | -39 | | -29.04 |
| Herbaceous | Root | Medium | -15.71 | -48.96 | | -39.08 |
| Herbaceous | Root | High | 0.82 | -10.22 | | -99.01 |
| Herbaceous | Root | Total | -13.79 | -40.11 | | -53.1 |


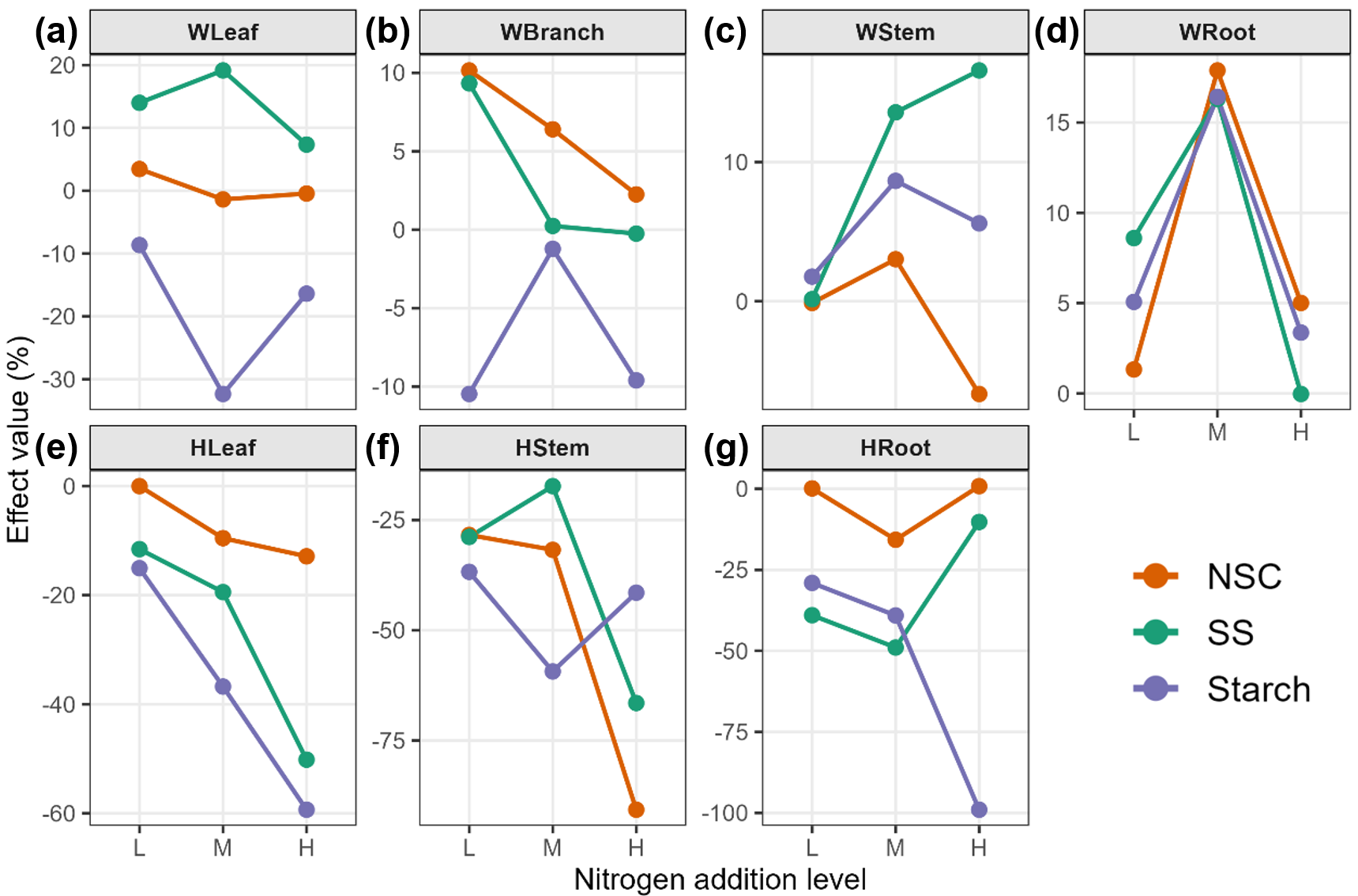


**Figure S3.** Effects values of different N enrichment on NSCs in different woody and herbaceous plant organs. The "W" before organ categories on the y-axis represents woody plants, and "H" represents herbaceous plants.

**Table S2** Effects values of different P enrichment on NSCs in different woody and herbaceous plant organs.

|  |  |  | Effect values (%) | |  |  |
| --- | --- | --- | --- | --- | --- | --- |
| Life forms | Organ | N level | NSC | Soluble sugar | | Starch |
| Woody | Leaf | Low | -7.9271 | -28.2401 | | -1.2662 |
| Woody | Leaf | Medium | -3.0649 | -15.3764 | | 13.2988 |
| Woody | Leaf | High | -10.5400 | -5.3020 | | -13.3215 |
| Woody | Leaf | Total | -10.4957 | -5.4720 | | -12.5818 |
| Woody | Branch | Low | 2.4591 | 0.1302 | | 5.4604 |
| Woody | Branch | Medium | 5.6467 | 11.8905 | | -1.5424 |
| Woody | Branch | High | 3.2656 | 3.9609 | | 3.0153 |
| Woody | Branch | Total | 3.7223 | 7.7188 | | 2.6077 |
| Woody | Stem | Low | 3.7003 | 2.4866 | | 5.9755 |
| Woody | Stem | Medium | -25.9150 | -11.9156 | | -59.6942 |
| Woody | Stem | High | 15.0742 | 27.3038 | | 11.5010 |
| Woody | Stem | Total | 13.6518 | 14.4965 | | 11.3983 |
| Woody | Root | Low | 2.4369 | 18.8538 | | -6.5513 |
| Woody | Root | Medium | 3.8253 | 0.3377 | | 8.2609 |
| Woody | Root | High | 25.9859 | -32.2844 | | 31.9588 |
| Woody | Root | Total | 24.9193 | -12.5715 | | 30.1276 |
| Herbaceous | Leaf | Low | -9.7406 |  | |  |
| Herbaceous | Leaf | Medium | -17.9620 |  | |  |
| Herbaceous | Leaf | High | -12.7831 |  | |  |
| Herbaceous | Leaf | Total | -12.9491 |  | |  |
| Herbaceous | Stem | Low | -8.7387 |  | |  |
| Herbaceous | Stem | Medium | 15.5213 |  | |  |
| Herbaceous | Stem | High | 20.9792 |  | |  |
| Herbaceous | Stem | Total | 15.9641 |  | |  |
| Herbaceous | Root | Low |  |  | |  |
| Herbaceous | Root | Medium |  |  | |  |
| Herbaceous | Root | High |  |  | |  |
| Herbaceous | Root | Total |  |  | |  |


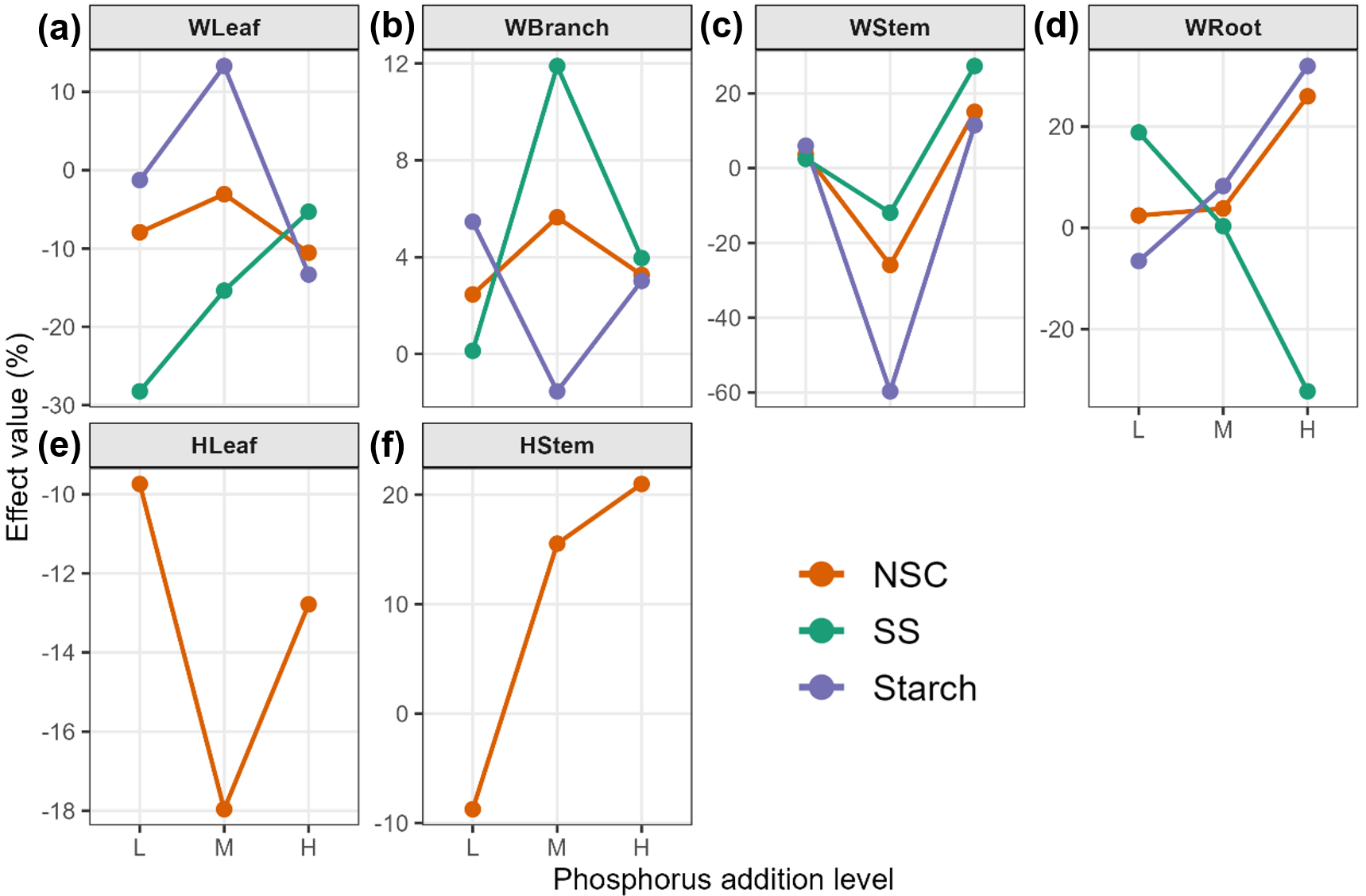


**Figure S4.** Effects values of different P enrichment on NSCs in different woody and herbaceous plant organs. The "W" before organ categories on the y-axis represents woody plants, and "H" represents herbaceous plants. Data for soluble sugar (SS) and starch in herbaceous plants (panels e and f) were insufficient to perform a robust trend analysis of the duration effect and are therefore not shown.


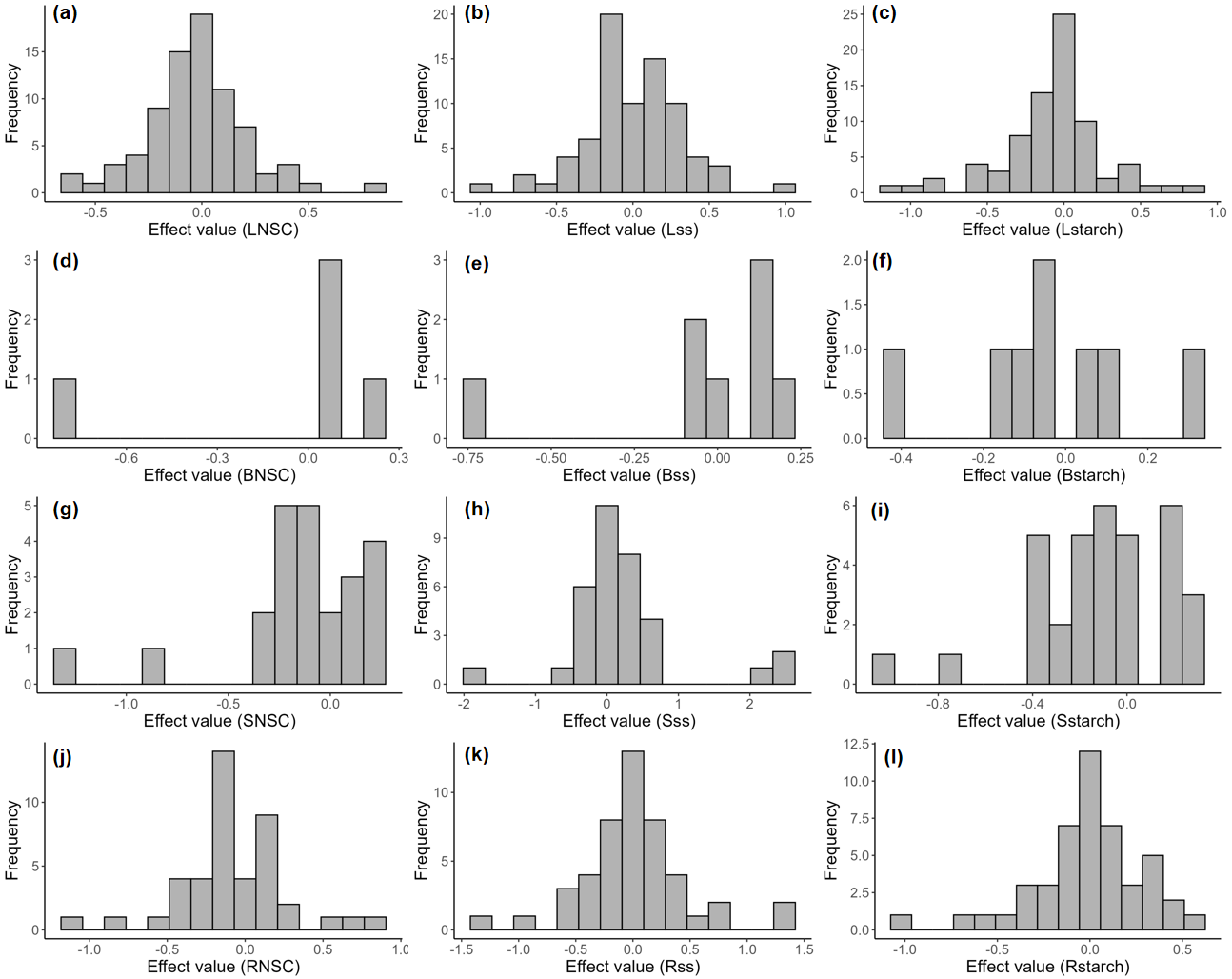


**Figure S5.** Distributions of meta-analysis effect sizes for different NSC components in response to nitrogen enrichment across plant organs.


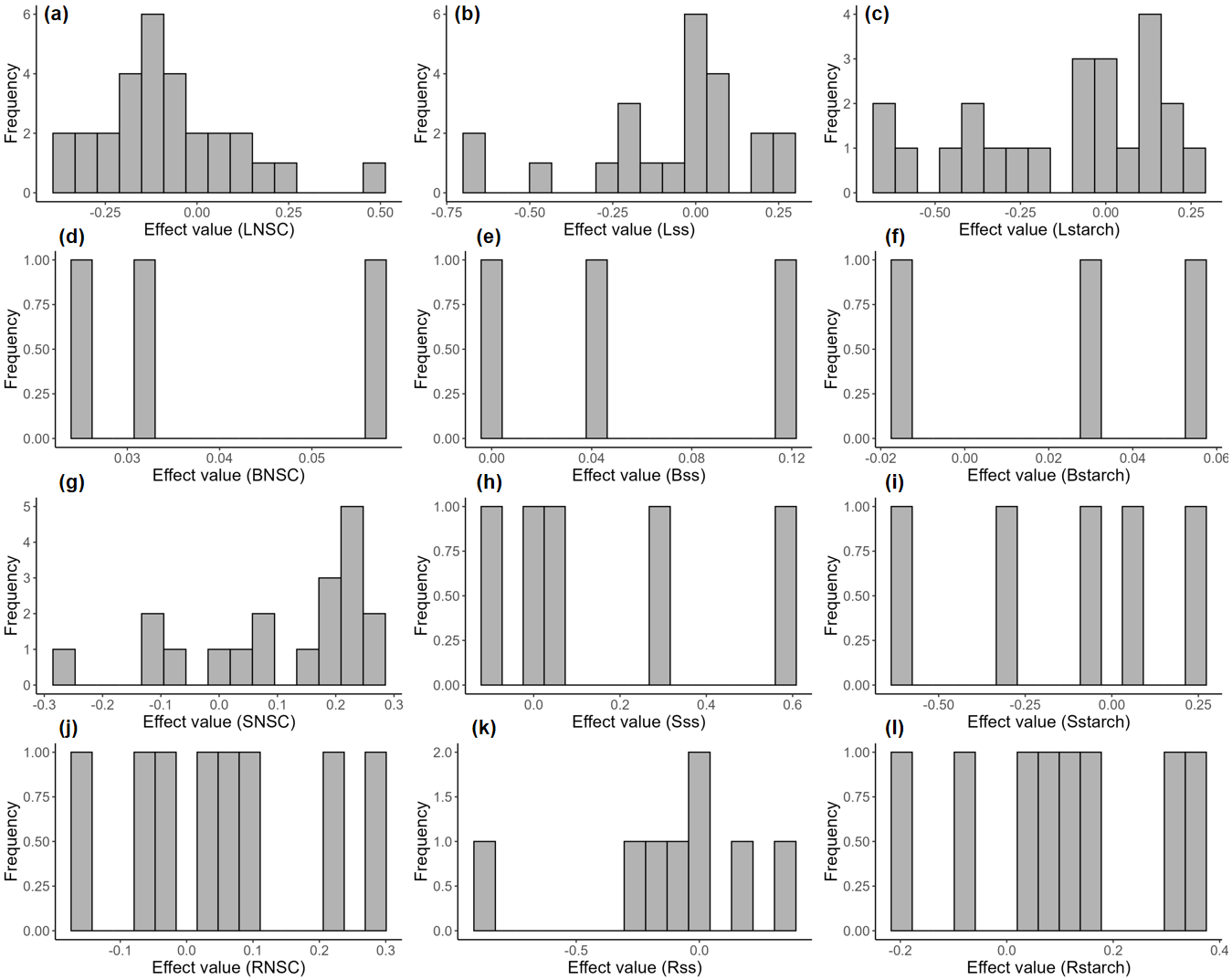


**Figure S6.** Distributions of meta-analysis effect sizes for different NSC components in response to phosphorus enrichment across plant organs.

**Data sources**

Ai, Z. M., Xue, S., Wang, G. L., & Liu, G. B. (2017). Responses of Non-structural Carbohydrates and C:N:P Stoichiometry of *Bothriochloa ischaemum* to Nitrogen Addition on the Loess Plateau, China. *Journal of Plant Growth Regulation*, *36*(3), 714-722. https://doi.org/10.1007/s00344-017-9673-y

Bu, W. S., Chen, F. S., Wang, F. C., Fang, X. M., Mao, R., & Wang, H. M. (2019). The species-specific responses of nutrient resorption and carbohydrate accumulation in leaves and roots to nitrogen addition in a subtropical mixed plantation. *Canadian Journal of Forest Research*, *49*(7), 826-835. https://doi.org/10.1139/cjfr-2018-0322

Chen, Y., Ke, M., Peng, Z., Yu, Y., & Mo, Q. (2021). Effects of nitrogen and phosphorus additions on concentrations of leaf non-structural carbohydrates,nitrogen,and phosphorus in Clerodendrum cyrtophyllum in a tropical forest. *Chinese Journal of Applied and Environmental Biology*, *27*(2), 389-397, Article 1006-687x(2021)27:2<389:Rdxtzw>2.0.Tx;2-d. <Go to ISI>://CSCD:6965345

Guo, D. L., Mitchell, R. J., & Hendricks, J. J. (2004). Fine root branch orders respond differentially to carbon source-sink manipulations in a longleaf pine forest. *Oecologia*, *140*(3), 450-457. https://doi.org/10.1007/s00442-004-1596-1

Ibrahim, M. H., Jaafar, H. Z. E., Rahmat, A., & Rahman, Z. A. (2011). Effects of Nitrogen Fertilization on Synthesis of Primary and Secondary Metabolites in Three Varieties of Kacip Fatimah (*Labisia Pumila* Blume). *International Journal of Molecular Sciences*, *12*(8), 5238-5254. https://doi.org/10.3390/ijms12085238

Jia, T., Fang, X. M., Yuan, Y., Fu, Y. X., Yi, M., Yuan, S. G., Guo, S. M., Lai, M., Xie, J. W., & Zhang, L. (2023). Phosphorus addition alter the pine resin flow rate by regulating tree growth and non-structural carbohydrates in a subtropical slash pine plantation. *Industrial Crops and Products*, *199*, Article 116782. https://doi.org/10.1016/j.indcrop.2023.116782

Jiang, X. Y., Song, M. Y., Qiao, Y. Q., Liu, M. Z., Ma, L., & Fu, S. L. (2022). Long-term water use efficiency and non-structural carbohydrates of dominant tree species in response to nitrogen and water additions in a warm temperate forest. *Frontiers in Plant Science*, *13*, Article 1025162. https://doi.org/10.3389/fpls.2022.1025162

Li, J. Y., Guo, Q. X., Zhang, J. X., Korpelainen, H., & Li, C. Y. (2016). Effects of nitrogen and phosphorus supply on growth and physiological traits of two *Larix* species. *Environmental and Experimental Botany*, *130*, 206-215. https://doi.org/10.1016/j.envexpbot.2016.06.006

Li, Q., Huang, Y., Zhou, D., & Cong, S. (2021). Mechanism of the trade-off between biological nitrogen fixation and phosphorus acquisition strategies of herbaceous legumes under nitrogen and phosphorus addition. *Chinese Journal of Plant Ecology*, *45*(3), 286-297, Article 1005-264x(2021)45:3<286:Trdltj>2.0.Tx;2-9. <Go to ISI>://CSCD:7000124

Li, R. S., Han, J. M., Zhu, L. Q., Zhao, L. J., Huang, X. L., Zhang, M. Y., Yang, Q. P., & Zhang, W. D. (2021). Does nitrogen fertilization impact nonstructural carbohydrate storage in evergreen *Podocarpus macrophyllus* saplings? *Journal of Forestry Research*, *32*(4), 1653-1661. https://doi.org/10.1007/s11676-020-01181-z

Li, Y. Y., Wang, Z. C., Liu, H. H., Zhang, C., Fu, S. L., & Fang, X. (2021). Responses in Growth and Anatomical Traits of Two Subtropical Tree Species to Nitrogen Addition, Drought, and Their Interactions. *Frontiers in Plant Science*, *12*, Article 709510. https://doi.org/10.3389/fpls.2021.709510

Liu, M. H., Wang, Y. X., Li, Q., Xiao, W. F., & Song, X. Z. (2019). Photosynthesis, Ecological Stoichiometry, and Non-Structural Carbohydrate Response to Simulated Nitrogen Deposition and Phosphorus Addition in Chinese Fir Forests. *Forests*, *10*(12), Article 1068. https://doi.org/10.3390/f10121068

Mo, Q. F., Chen, Y. Q., Yu, S. Q., Fan, Y. X., Peng, Z. T., Wang, W. J., Li, Z. A., & Wang, F. M. (2020). Leaf nonstructural carbohydrate concentrations of understory woody species regulated by soil phosphorus availability in a tropical forest. *Ecology and Evolution*, *10*(15), 8429-8438. https://doi.org/10.1002/ece3.6549

Ouyang, S. N., Tie, L. H., Saurer, M., Bose, A. K., Duan, H. L., Li, M. H., Xu, X. L., Shen, W. J., & Gessler, A. (2024). Divergent role of nutrient availability in determining drought responses of sessile oak and Scots pine seedlings: evidence from ^13^C and ^15^N dual labeling. *Tree Physiology*, *44*(1), Article tpad105. https://doi.org/10.1093/treephys/tpad105

Peng, Z. T., Chen, M. X., Huang, Z. J., Zou, H. R., Qin, X. L., Yu, Y. H., Bao, Y. T., Zeng, S. C., & Mo, Q. F. (2021). Non-Structural Carbohydrates Regulated by Nitrogen and Phosphorus Fertilization Varied with Organs and Fertilizer Levels in *Moringa oleifera* Seedlings. *Journal of Plant Growth Regulation*, *40*(4), 1777-1786. https://doi.org/10.1007/s00344-020-10228-8

Shang, J., Gao, T., Wang, W., Zhou, X., & Zong, Y. (2022). Effect of Nitrogen Addition for Two Consecutive Years on Photosynthetic Characteristics, Carbon and Nitrogen Distribution of Populus * euramericana “Zhongjin7” Seedlings. *Scientia Silvae Sinicae*, *58*(6), 23-32, Article 1001-7488(2022)58:6<23:Lx2ndt>2.0.Tx;2-w. <Go to ISI>://CSCD:7314465

Wang, F. C., Chen, F. S., Wang, G. G., Mao, R., Fang, X. M., Wang, H. M., & Bu, W. S. (2019). Effects of Experimental Nitrogen Addition on Nutrients and Nonstructural Carbohydrates of Dominant Understory Plants in a Chinese Fir Plantation. *Forests*, *10*(2), Article 155. https://doi.org/10.3390/f10020155

Wang, J., Guan, X., Zhang, W., Huang, K., Zhu, M., & Yang, Q. (2021). Responses of biomass allocation patterns to nitrogen addition of Cunninghamia lanceolata seedlings. *Chinese Journal of Plant Ecology*, *45*(11), 1231-1240, Article 1005-264x(2021)45:11<1231:Smymsw>2.0.Tx;2-e. <Go to ISI>://CSCD:7146353

Wang, K., Lei, H., Xia, Y., & Yu, G. Q. (2017). Responses of non-structural carbohydrates of poplar seedlings to increased precipitation and nitrogen addition.. *Ying yong sheng tai xue bao = The journal of applied ecology*, *28*(2), 399-407. https://doi.org/10.13287/j.1001-9332.201702.012

Wang, R. Z., Yun, L. L., Mao, Y. X., Yan, T. W., Wei, W. J., You, W. Z., & Zhang, H. D. (2024). Nitrogen deposition alters drought-induced changes in biomass and nonstructural carbohydrates allocation patterns of *Quercus mongolica* seedlings. *Scientia Horticulturae*, *325*, Article 112573. https://doi.org/10.1016/j.scienta.2023.112573

Wang, X. Y., Schoenbeck, L., Gessler, A., Yang, Y., Rigling, A., Yu, D. P., He, P., & Li, M. H. (2022). The effects of previous summer drought and fertilization on winter non-structural carbon reserves and spring leaf development of downy oak saplings. *Frontiers in Plant Science*, *13*, Article 1035191. https://doi.org/10.3389/fpls.2022.1035191

Wei, N., Li, G., Cai, M., Shi, W., Liu, W., Xue, L., & Li, J. (2021). Effects of slow-release fertilization rates on seedling quality and field survival rates of four exotic oaks. *Journal of Nanjing Forestry University. Natural Sciences Edition*, *45*(3), 53-60, Article 1000-2006(2021)45:3<53:Hsfsdl>2.0.Tx;2-g. <Go to ISI>://CSCD:6982729

Xie, T. T., Shan, L. S., & Zhang, W. T. (2022). N addition alters growth, non-structural carbohydrates, and C:N:P stoichiometry of *Reaumuria soongorica* seedlings in Northwest China. *Scientific Reports*, *12*(1), Article 15390. https://doi.org/10.1038/s41598-022-19280-8

Xu, Y., Xu, K., Wang, W., Yu, S., Ruan, H., Ge, Z., Wang, G., & Han, Q. (2014). The response of carbohydrates compositions in fine root of poplar at different ages to nitrogen depositions. *Journal of Nanjing Forestry University. Natural Sciences Edition*, *38*(3), 13-18, Article 1000-2006(2014)38:3<13:Btllys>2.0.Tx;2-x. <Go to ISI>://CSCD:5146440

Yan, T., Wang, L. Y., Wang, P. L., & Zhong, T. Y. (2023). Stability in the leaf functional traits of understory herbaceous species after 12-yr of nitrogen addition in temperate larch plantations. *Frontiers in Plant Science*, *14*, Article 1282884. https://doi.org/10.3389/fpls.2023.1282884

Zhang, D., Jing, H., & Wang, G. L. (2019). Responses of non-structural carbohydrates content in leaves of different plant species in Pinus tabuliformis plantation to nitrogen addition. *Ying yong sheng tai xue bao = The journal of applied ecology*, *30*(2), 489-495. https://doi.org/10.13287/j.1001-9332.201902.022

Zhang, H. X., Yuan, F. H., Wu, J. B., Jin, C. J., Pivovaroff, A. L., Tian, J. Y., Li, W. B., Guan, D. X., Wang, A. Z., & McDowell, N. G. (2021). Responses of functional traits to seven-year nitrogen addition in two tree species: coordination of hydraulics, gas exchange and carbon reserves. *Tree Physiology*, *41*(2), 190-205. https://doi.org/10.1093/treephys/tpaa120

Zhang, W., Shan, L., Li, Y., Bai, Y., & Ma, J. (2020). Effects of nitrogen addition and precipitation change on non-structural carbohydrates in Reaumuria soongorica seedlings. *Chinese Journal of Ecology*, *39*(3), 803-811, Article 1000-4890(2020)39:3<803:Dtjyjy>2.0.Tx;2-o. <Go to ISI>://CSCD:6670500

Zhang, W. Y., Gong, J. R., Zhang, Z. H., Song, L. Y., Lambers, H., Zhang, S. Q., Dong, J. J., Dong, X. D., & Hu, Y. X. (2023). Soil phosphorus availability alters the correlations between root phosphorus-uptake rates and net photosynthesis of dominant C_3_ and C_4_ species in a typical temperate grassland of Northern China. *New Phytologist*, *240*(1), 157-172. https://doi.org/10.1111/nph.19167

Zheng, W. H., Li, R. S., Yang, Q. P., Zhang, W. D., Huang, K., Guan, X., Chen, L. C., Yu, X., Wang, Q. K., & Wang, S. L. (2023). Allocation patterns of nonstructural carbohydrates in response to CO_2_ elevation and nitrogen deposition in *Cunninghamia lanceolata* saplings. *Journal of Forestry Research*, *34*(1), 87-98. https://doi.org/10.1007/s11676-022-01533-x
